# Prähospitale Bluttransfusion: Chancen und Herausforderungen für den deutschen Rettungsdienst

**DOI:** 10.1007/s00101-024-01463-9

**Published:** 2024-10-02

**Authors:** Jens Schwietring, Dirk Wähnert, Lucas Sebastian Scholl, Karl-Christian Thies

**Affiliations:** 1 Ruhr-Universität Bochum, Medizinische Fakultät, Bochum, Deutschland; 2 ADAC Luftrettung gGmbH, Hansastr. 19, 80686 München, Deutschland; 3 Universität Bielefeld, Medizinische Fakultät und Universitätsklinikum OWL, Ev. Klinikum Bethel, Universitätsklinik für Unfallchirurgie und Orthopädie, Bielefeld, Deutschland; 4 Universität Bielefeld, Bielefeld, Deutschland; 5 Universität Bielefeld, Medizinische Fakultät und Universitätsklinikum OWL, Ev. Klinikum Bethel, Universitätsklinik für Anästhesiologie, Intensivmedizin, Notfallmedizin, Transfusionsmedizin und Schmerztherapie, Bielefeld, Deutschland

**Keywords:** Notfall Transfusion, Haemorrhagischer Schock, Massiv Transfusion, Blutkomponenten, Outcome, Emergency transfusion, Haemorrhagic shock, Massive transfusion, Blood components, Outcome

## Abstract

**Hintergrund:**

Blutverlust ist die Hauptursache potenziell vermeidbarer Todesfälle bei schweren Verletzungen. Behandlungsprioritäten sind die sofortige Kontrolle der Blutung und die Transfusion von Blutprodukten zur Aufrechterhaltung des Sauerstofftransports und zur Therapie der traumainduzierten Koagulopathie. Während die prähospitale Transfusion von Blutprodukten (PHBT) in unseren Nachbarländern etabliert ist, hat die fragmentierte Struktur der Rettungsdienste die Einführung von PHBT-Programmen in Deutschland verzögert. Unsere Arbeit bietet eine aktuelle Perspektive auf die Entwicklung, internationale Praktiken und den Forschungsbedarf zur Anwendung von PHBT im deutschen Kontext.

**Methodik:**

Diese narrative Übersicht basiert auf einer PubMed-Suche mit den Schlüsselwörtern „prehospital“ und „blood*“. Von 4738 gefundenen Artikeln bezogen sich 333 auf PHBT und wurden einer weiteren detaillierten Sichtung unterzogen. Die Literatur, einschließlich zitierter Studien, wurde in Bereiche wie Geschichte, Rationale, internationale Praktiken und Evidenz kategorisiert und entsprechend ihrer Qualität in die Auswertung einbezogen.

**Ergebnisse:**

Der Nutzen der frühzeitigen Bluttransfusion bei schwerem Trauma ist seit dem Ersten Weltkrieg belegt, was die Bestrebungen erklärt, diese lebensrettende Maßnahme schon im prähospitalen Bereich einzuleiten. Neuere randomisierte Studien, die aufgrund der komplexen Fragestellung mit Design- und Rekrutierungsproblemen kämpften, haben widersprüchliche Ergebnisse hinsichtlich des Überlebensvorteils im zivilen Bereich geliefert. Die Lehren aus diesen Arbeiten lassen bezweifeln, ob randomisierte Studien tatsächlich in der Lage sind, Fragen zum Überlebensvorteil zu klären. Trotz der genannten Schwierigkeiten gibt es einen erkennbaren Trend, der bei transfundierten Patienten auf Verbesserungen des Outcome hindeutet. In Deutschland beträgt die Inzidenz des traumaassoziierten Schocks 38/100.000 Einwohner pro Jahr. Geschätzt wird, dass zwischen 300 und 1800 Patienten jährlich von PHBT profitieren könnten.

**Schlussfolgerungen:**

Die prähospitale Gabe von Blutprodukten erscheint vielversprechend, doch bleibt offen, welche Patientengruppen davon profitieren und welche Blutprodukte am besten geeignet sind. In Deutschland sind PHBT-Programme noch nicht weit verbreitet. Paradoxerweise bietet diese Situation, zusammen mit dem umfangreichen Trauma Register der Deutschen Gesellschaft für Unfallchirurgie, eine hervorragende Ausgangssituation für umfassende prospektive Kohortenstudien, um Patienten-Outcome, Logistik und Implementierungsstrategien zu untersuchen. Solche Studien könnten helfen, evidenzbasierte PHBT-Richtlinien auszuarbeiten und in deutsche Traumaversorgungsprotokolle zu integrieren.

## Abstract (en)

**Background:**

Exsanguination is the leading cause of preventable death in severe trauma. Immediate hemorrhage control and transfusion of blood products are critical to maintain oxygen delivery and address trauma-induced coagulopathy. While prehospital blood product transfusion (PHBT) is established in neighboring countries, the fragmented configuration of Germany’s emergency medical service (EMS) infrastructure has delayed the adoption of widespread PHBT programmes. This review aims to provide an updated perspective on the evolution, international practices and research needs of PHBT within the German context.

**Methods:**

This narrative review is based on a PubMed search using the search terms “prehospital” and “blood*”. From an initial 4738 articles, 333 were directly related to PHBT and were subjected to further detailed examination. The literature, including referenced studies, was categorized into areas such as history, rationale, international practices, and evidence, and analyzed for quality.

**Results:**

The benefit of early blood transfusion in major trauma has been established since WW1, explaining the efforts to initiate this lifesaving intervention as early as possible in the care pathway, including the prehospital field. Recent randomized trials have faced design and recruitment challenges, reflecting the complexity of the research question. These trials have yielded inconclusive results regarding the survival benefits of PHBT in civilian settings. This scenario raises doubts about the capability of randomized trials to resolve questions concerning survival advantages. Despite these difficulties, there is a discernible trend indicating potential improvements in patient outcomes. In Germany, the incidence of trauma-associated shock stands at 38 per 100,000 individuals per year. It is estimated that between 300 and 1800 patients annually possibly benefit from PHBT.

**Conclusion:**

Prehospital Blood Transfusion appears to be promising but identifying patient groups most likely to benefit as well as the most suitable blood products remain unresolved issues. In Germany PHBT programs are not yet widely established. Paradoxically, this situation, paired with the extensive German Trauma Registry, provides a prime opportunity for comprehensive prospective cohort studies, addressing the balance between PHBT benefits, logistical feasibility, and implementation strategies. Such studies are essential for establishing guidelines and integrating PHBT efficiently into German trauma care protocols.

## Einleitung

Die Hauptursache vermeidbarer Todesfälle bei schweren Verletzungen ist das Verbluten [1, 2]. Behandlungsprioritäten sind die sofortige Kontrolle der Blutung und die Transfusion von Blutprodukten zur Aufrechterhaltung des Sauerstofftransports und zur Therapie der traumainduzierten (TIC) Koagulopathie. Europäische Leitlinien [3] empfehlen, Schwerverletzte im hämorrhagischen Schock direkt in überregionale Traumazentren zu transportieren, um eine optimale Behandlung so schnell wie möglich sicherzustellen. In Großbritannien hat diese Praxis tatsächlich die 30-Tage-Mortalität von Schwerverletzten um 25 % gesenkt [4]. Allerdings bedingt der direkte Transport in überregionale Traumazentren oft lange Transportwege. Daher werden nun auch in europäischen Ländern zunehmend präklinische Blutprodukte zur Überbrückung eingesetzt [5]. Jedoch werden die Vor- und Nachteile präklinischer Transfusionen kontrovers diskutiert, da die wissenschaftliche Evidenz uneinheitlich ist [6].

In diesem Artikel geben wir einen Überblick über die Entwicklung der prähospitalen Gabe von Blutprodukten, beschreiben die internationale Praxis, analysieren die zugrunde liegende wissenschaftliche Evidenz und diskutieren den möglichen Nutzen im Kontext des deutschen Rettungssystems sowie den weiterhin bestehenden Forschungsbedarf.

## Methode

Grundlage dieser narrativen Übersichtsarbeit ist eine PubMed-Literaturrecherche mit den Suchtermen „prehospital“ und „blood*“. Diese ergab mit Suchdatum 18.02.24 insgesamt 4738 Artikel. Die Ergebnisse wurden zuerst nach Titeln gesichtet. Hiervon hatten 333 Artikel einen direkten Bezug zur prähospitalen Transfusion von Blutprodukten („prehospital blood transfusion“, PHBT) und wurden einer Sichtung der Abstracts unterzogen.

Originalarbeiten, Reviews, Editorials sowie Fallserien wurden in die Auswertung einbezogen. Zusätzlich verfolgten wir die in den gefundenen Artikeln zitierten Referenzen, um die Vollständigkeit der Recherche zu gewährleisten. Die Inhalte haben wir den Themengruppen Geschichte, Rationale, internationale Praxis, Indikationsstellung, angewendete Produkte, Patientensicherheit, Logistik und Evidenz zugeordnet. Dabei wurden die einzelnen Studien nach ihrer Qualität gewichtet und entsprechend in unsere Analyse einbezogen.

### Geschichte

Die Verwendung von Bluttransfusionen in der Versorgung von Patienten im hämorrhagischen Schock hat eine lange Geschichte, aber erst die Entdeckung der AB0-Blutgruppen durch Karl Landsteiner im Jahre 1901 legte den Grundstein für die Einführung der Bluttransfusion in die klinische Praxis. Durchgesetzt hat sich die frühzeitige Transfusionsbehandlung bei Schwerverletzten erstmals in der alliierten Militärmedizin während des Ersten Weltkriegs [7]. Obwohl die Möglichkeiten damals beschränkt waren, wurde der Mehrwert der frühen Transfusion von Vollblut schnell erkannt. Parallel dazu wurde die gängige Praxis der Gabe großer Mengen Kochsalzlösung zur Volumenwiederherstellung bei hämorrhagischem Schock infrage gestellt [8]. Die Einrichtung der ersten Blutbank in Vorbereitung auf die Schlacht von Ypern erlaubte erstmalig die Transfusion von Zitratblutkonserven in größerem Umfang [9]. Im Zweiten Weltkrieg gelangten bei der US-Armee anfänglich mehr Albumin, lyophilisiertes Plasma und Frischplasma zur Anwendung [10]. Erst gegen Mitte des Krieges wurden wieder vermehrt Warmblut und Zitratvollblutkonserven eingesetzt [11]. Auf der sowjetischen Seite wurden sogar erfolgreiche präklinische Reanimationen unter Einsatz von Vollblut beschrieben [12].

Die Anwendung der Transfusionsbehandlung nahe der Frontlinie wurden im Korea- und Vietnamkrieg noch erheblich ausgeweitet, wobei sogar erfolgreiche Massivtransfusionen durchgeführt wurden [2]. Die positiven Erfahrungen mit präklinischer Blutgabe aus den späteren Irak- und Afghanistankriegen [13] resultieren aus den kriegsspezifischen Verletzungsmustern; massiver Blutverlust war mit über 90 % die führende, potenziell behandelbare Todesursache auf dem Schlachtfeld [14]. Diese Kriegserfahrungen beeinflussten im Laufe der Zeit zunehmend auch die zivile Praxis, und heutzutage stehen präklinische Blutprodukte in einigen europäischen Ländern rund um die Uhr und flächendeckend zur Verfügung [15].

### Damage Control Resuscitation – die Rationale

Die Hälfte aller potenziell behandelbaren Ursachen prähospitaler Todesfälle nach Trauma im zivilen Bereich ist auf Verblutung zurückzuführen [1, 16]. Darüber hinaus können schwere Verletzungen, die mit hämorrhagischem Schock einhergehen, die gengesteuerte Freisetzung proinflammatorischer Mediatoren triggern und die „lethal triad“ oder „tödliche Trias“ auslösen; den Teufelskreis aus Hypothermie, metaboler Acidose und Koagulopathie. Die TIC kann durch Hyperfibrinolyse zum Verbluten beitragen und sekundär bei Hypofibrinolyse ins Multiorganversagen führen [17]. Die traditionelle Anwendung großer Mengen Kristalloide zur Initialbehandlung des hämorrhagischen Schocks ist problembehaftet; sie kann Ödeme, abdominelles Kompartmentsyndrom, ARDS sowie Verdünnungskoagulopathie verursachen und ist zudem mit erhöhter Mortalität assoziiert [18]. Bei schwer verletzten, transfusionsbedürftigen Patienten mit nicht unmittelbar kontrollierbaren Blutungen hat das „Damage-Control-Resuscitation“(DCR)- oder „Haemostatic-Resuscitation“-Konzept den „liberalen“ Volumenersatz abgelöst [19, 20]. DCR beruht auf der Vermeidung von Kristalloiden, temporärer permissiver Hypotension sowie der unmittelbaren Gabe von Blutprodukten, mit den Zielen, einerseits den akuten Blutverlust zu minimieren, andererseits die Gerinnung zu stabilisieren, um das Einsetzen der tödlichen Trias zu verhindern [21].

Retrospektive Untersuchungen aus der Militärmedizin belegen, dass bei schwer verletzten Patienten die Sterblichkeit mit der Dauer bis zur ersten Transfusion signifikant steigt [22]. Im zivilen Sektor scheint sich diese Beobachtung trotz unterschiedlicher Verletzungsmuster zu bestätigen [23, 24]. Dies unterstreicht die Bestrebungen, Blutprodukte auch im zivilen Bereich schon in der Prähospitalphase anzuwenden.

### Internationale Praxis

Im Gegensatz zu anderen europäischen Ländern (Frankreich, Skandinavien, UK, Niederlande) und den USA hat sich die prähospitale Gabe von Blutprodukten in Deutschland noch nicht durchgesetzt und findet erstmalig in den S3-Leitlinien der Deutschen Gesellschaft für Unfallchirurgie (DGU) zur Schwerverletztenversorgung aus dem Jahr 2022 Erwähnung [25]. Derzeit sind prähospitale Transfusionsprogramme in Deutschland im Aufbau begriffen. Blutprodukte stehen jedoch nicht regelhaft für die präklinische Anwendung zur Verfügung. Beachtlich ist die große Diskrepanz zwischen Deutschland und unseren Nachbarländern. Während 89 % der französischen Rettungsdienste Blutprodukte mitführen oder Protokolle zum notfallmäßigen Transport von Blutprodukten an die Einsatzstelle vorhalten, ist dies in Deutschland nur bei 10 % der Luftrettungsstandorte der Fall [26]. Führten in Großbritannien (UK) im Jahre 2016 noch 64 % der Luftrettungsdienste Blutprodukte mit [27], sind inzwischen fast alle Rettungshubschrauber mit Blutprodukten ausgerüstet [28]. In den USA reicht die Praxis der prähospitalen Bluttransfusion im zivilen Luftrettungsdienst über 40 Jahre zurück [29]. Auch in Kanada werden prähospitale Bluttransfusionen im Luftrettungsdienst durchgeführt und mehr als ein Drittel der Dienste führt Blutprodukte mit [30]. In Rettungsdienstbereich Sydney, Australien, ist die prähospitale Gabe von Erythrozytenkonzentraten seit mehr als 10 Jahren etabliert [31].

Im militärischen Kontext ist der Nutzen der prähospitalen Bluttransfusion unstrittig. Bei der US-Armee ist die prähospitale Verwendung von Blutprodukten seit dem Vietnamkrieg fest etabliert [22] und durch klinische Richtlinien untermauert [32].

Hieraus abgeleitet sind die Leitlinien der Vereinigung der US-amerikanischen Blutbanken für den zivilen Einsatz von prähospitalen Blutprodukten [33]. Seit dem Irakkrieg haben auch die britischen Streitkräfte die Transfusion von Blutprodukten in ihr prähospitales Versorgungskonzept der „forward resuscitation“ integriert [34, 35], und es gibt wenige Zweifel unter Militärmedizinern, dass die PHBT dazu beigetragen hat, die Sterblichkeit auf dem Schlachtfeld in den letzten 20 Jahren dramatisch zu senken [36].

### Transfusionstrigger

Eine Vielzahl von Publikationen widmet sich dem Thema prähospital anwendbarer Transfusionstrigger, die es dem aufnehmenden Krankenhaus ermöglichen sollen, vor Eintreffen des Patienten eine Massivtransfusion vorzubereiten. In einer lesenswerten systematischen Übersichtsarbeit zu diesem Thema [37] wurden 52 verschiedene Scores identifiziert und miteinander verglichen. Die am häufigsten verwendeten Kriterien zur Einschätzung des hämorrhagischen Schocks sind Hypotonie (in der Regel systolischer Blutdruck unter 90 mm Hg) und Tachykardie (100–120 bpm) sowie der Bewusstseinszustand des Patienten, das Verletzungsmuster, das Vorliegen einer aktiven Blutung, v. a. aber die subjektive Einschätzung des behandelnden Klinikers [38]. Darüber hinaus fließen auch die Ergebnisse von „point of care tests (PoCT)“ wie Basendefizit, Lactat, Hämoglobinwert und Ultraschallbefund in unterschiedlicher Gewichtung in die Entscheidung ein. Die aus diesen Parametern abgeleiteten Scores sind allerdings in der Regel für den innerklinischen Bereich entwickelt und nicht für die präklinische Entscheidungsfindung validiert; aus dem Bedarf einer Massivtransfusion nach Krankenhausaufnahme wird auf den möglichen Nutzen einer prähospitalen Transfusion geschlossen. Bislang wurde im klinischen Kontext die Massivtransfusion als eine Transfusionsmenge von ≥ 10 Erythrozytenkonzentraten (EK) innerhalb von 24 h definiert. Alternativ zeigt die Verwendung einer Definition, die eine Transfusionsmenge von 3–4 EK innerhalb einer Stunde beschreibt, ein besseres Evaluationsergebnis [39]. Es ist anzunehmen, dass diese neue Definition am ehesten die Patientengruppe abbildet, die von einer prähospitalen Transfusion profitiert.

**Abb. 1:**
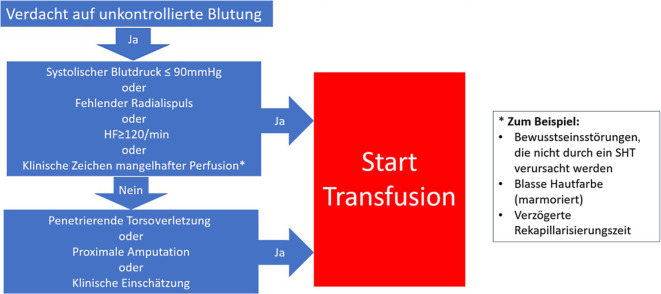
Entscheidungshilfe zum Einsatz prähospitaler Erythrozytenkonzentrate der Luftrettung Amsterdam. (Mod. nach [38])

Die an Daten des Traumaregisters der Deutschen Gesellschft für Unfallchirurgie (DGU) extern validierten Scores TASH (Trauma Associated Hemorrhage Score) [40, 41] und mTICCS (modified Trauma Induced Coagulopathy Clinical Score) [5, 42–45] scheinen für den deutschen Raum am ehesten geeignet zu sein, haben sich aber vermutlich wegen ihrer Komplexität in der Praxis nicht durchgesetzt. Man darf annehmen, dass prähospitales PoCT die Validität von Scoring-Systemen verbessert, jedoch ist dies bislang nicht abschließend bestätigt [46, 47]. Als praktisches Beispiel einer Entscheidungshilfe zur PHBT findet sich in der Abb. 1 das Vorgehen der Kollegen aus dem niederländischen Luftrettungssystem.

### Welche Produkte werden eingesetzt?

*Erythrozytenkonzentrate* (EK) der Blutgruppe 0 Rh-negativ werden bevorzugt für Transfusionen im Rettungsdienst verwendet. In Europa hat diese Blutgruppe jedoch nur eine Prävalenz von 3–6 %. Demzufolge ist die Zahl der verfügbaren Spender und somit Konserven sehr begrenzt, weshalb häufig auf 0 Rh-positive EK zurückgegriffen werden muss. Wird im Notfall bei unbekannter Blutgruppe Blut der Gruppe 0 Rh-positiv transfundiert, liegt die Alloimmunisierungsrate unter 4 %. Die Alloimmunisierungsrate steigt jedoch auf 17 %, wenn aufgrund von Mangel an 0 Rh-negativen Konserven Patienten mit dieser Blutgruppe 0 Rh-positive EK erhalten müssen. Aus diesem Grund macht es Sinn, 0 Rh-negative Konserven im elektiven Klinikbetrieb einzusetzen und 0 Rh-positive Konserven bei lebensbedrohlichen Notfällen bei Patienten mit unbekannter Blutgruppe wie z. B. im Rettungsdienst zu verwenden [48].

*Plasma* kommt entweder als aufgetautes Frischplasma (FFP) oder als lyophilisiertes Plasma im Rettungsdienst zur Anwendung. Aufgetautes Frischplasma ist bis zu 7 Tage verwendbar, wenn die Kühlkette mit Temperaturen zwischen 2 und 6^o^C eingehalten wird. Die Faktoraktivität fällt in diesem Zeitraum zwar signifikant ab, bleibt allerdings bis auf Faktor VIII (56 %) und Protein S (51 %) im Referenzbereich. Der Faktorenabfall wird als nicht klinisch relevant betrachtet, solange das Plasma nicht zu Massivtransfusionen herangezogen wird [49]. Allerdings hat das Patientenkollektiv, das von einer präoperativen Bluttransfusion (PHBT) profitieren könnte, wohl auch eine erhöhte Wahrscheinlichkeit, eine Massivtransfusion zu benötigen. Traditionell wird FFP der Blutgruppe AB als Universalspender verwendet. Diese Blutgruppe hat allerdings in Deutschland nur eine Prävalenz von 4 % und ist somit ebenfalls eine sehr knappe Ressource. Daher wird häufiger auch Plasma der Blutgruppe A eingesetzt. Unabhängig vom Anti-B-Titer scheint dies genauso gut verträglich wie AB-Plasma zu sein; ein erhöhtes Auftreten von Unverträglichkeitsreaktionen wurde nicht beobachtet [50].

Alternativ zu Frischplasma wird zusehends lyophilisiertes Plasma eingesetzt. Durch den Blutspendedienst West des Deutschen Roten Kreuz (DRK) wird das Präparat Lyo-plasN‑w als Produkt von Einzelspendern aber auch als LyoPlas P in einer gepoolten Version angeboten.

Die Faktoraktivität hängt von der Lagerdauer und der Lagertemperatur ab. Bei 25^o^C zeigt das Produkt zwar einen signifikanten Abfall der Faktoraktivität, die allerdings nach einer Lagerzeit von 12 Monaten immer noch im unteren Normbereich liegt [51]. In einer französischen Untersuchung führte lyophilisiertes Plasma zu einem schnelleren, ausgeprägteren und längeren Anstieg der Fibrinogenkonzentrationen und einer Verbesserung der Koagulopathie im Vergleich zu FFP bei der initialen Behandlung von Traumapatienten [52]. Im prähospitalen Einsatz scheint lyophilisiertes Plasma gegenüber FFP zumindest gleichwertig zu sein, bietet jedoch v. a. im Rettungsdienst erhebliche logistische Vorteile [53].

*Fibrinogen *gelangt im Rettungsdienst in der Form von Plasma, Kryopräzipitat und als Faktorkonzentrat zur Anwendung. Faktorkonzentrate müssen entsprechend den Herstellervorgaben entweder bei 2–8^o^C oder bei Temperaturen ≤ 25^o^C gelagert werden. Fibrinogenmangel, ein Hauptmerkmal der traumaassoziierten Koagulopathie [54], ist ein unabhängiger Prädiktor für Mortalität bei Schwerverletzten mit hohem Blutverlust [55]. Eine retrospektive Untersuchung weist auf eine signifikante Mortalitätssenkung durch frühzeitige Fibrinogensubstitution bei traumabedingten lebensbedrohlichen Blutungen hin [56].

Für die prähospitale Anwendung gibt die deutsche S3-Leitlinie Polytrauma für die nichtbeherrschbare Blutung vor, dass die Gabe von Fibrinogen nach der Applikation von Tranexamsäure erwogen werden kann [25].

Laut Empfehlungen des Nationalen Gesundheitsdienstes New South Wales (Australien) könnte in Ermangelung von Plasmapräparaten Fibrinogenkonzentrat als sinnvolle präklinische Alternative in Betracht gezogen werden [57]. Diese Empfehlung ist allerdings im Lichte der besonders langen Transportwege in Australien zu betrachten.

In den 1970er-Jahren wurde die Behandlung mit *Vollblutkonserven* durch die Blutkomponententherapie ersetzt, da sie eine effizientere, sicherere und optimierte Bereitstellung von Blutprodukten ermöglichte. Neuere positive Erfahrungen mit Vollblut der Blutgruppe 0 mit niedrigem Antikörpertiter (‚Low Titer O Whole Blood‘, LTOWB) aus dem Militärbereich haben jedoch auch im zivilen Bereich zu einer Renaissance geführt. Das wieder erwachte Interesse an Vollblut für Massivtransfusionen resultiert aus seiner physiologisch ausgewogenen Zusammensetzung, einer vereinfachten Logistik bei massivem Blutverlust, einem potenziell geringeren Risiko für Komplikationen wie Transfusion Related Acute Lung Injury (TRALI) und verbesserten Sicherheitstests für LTOWB. Mehrere retrospektive Untersuchengen, insbesondere aus dem US-Trauma-Register, haben gezeigt, dass die Verwendung von Vollblut bei Massivtransfusionen unabhängig von der Empfängerblutgruppe mit einer signifikanten Verringerung der Mortalität einhergeht, insbesondere bei Patienten mit hohem Mortalitätsrisiko [58–62]. Inzwischen wird LTOWB in 42 % der US-amerikanischen Traumazentren in der Notfallversorgung eingesetzt [63]. Die französische T‑STORHM-Studie [64] sowie die britische SWIFT-Studie [65, 66] untersuchen derzeit, ob die Vorteile von Vollblut auch in der präklinischen Anwendung in randomisierten Studien bestätigt werden können. Im norwegischen Luftrettungsdienst wird LTOWB bereits seit mehreren Jahren routinemäßig angewendet [67]. In Summe lässt sich festhalten, dass auch die im zivilen, amerikanischen Bereich erfassten Ergebnisse der retrospektiven [18] und prospektiven [59] Studien einen Mortalitätsvorteil zeigen. Diesbezüglich hat die Arbeitsgruppe Trauma Hemostatsis and Oxygenation Research (THOR) der Vereinigung der US-amerikanischen Blutbanken [33] in ihren „Best Practice Guidelines“ die Gabe von Vollblut (Infobox 1) im Prähospitalbereich mitaufgenommen.

Die Richtlinien der US Armee sehen sogar eine Prioritätenliste beim Einsatz bestimmter Produkte vor: 1) gelagertes LTOWB, 2) frisches LTOWB von voruntersuchten Spendern, 3) Plasma, EK und Thrombozytenkonzentrate im Verhältnis, 1:1:1 4) Plasma und EK im Verhältnis 1:1, 5) nur Plasma oder nur EK [32].

Tab. 1 vergleicht die Indikationen und Lagerungsmerkmale von Blutprodukten hinsichtlich ihrer präklinischen Verwendung.

**Tab. 1:** Indikationen und Lagerungsmerkmale von Blutprodukten für den präklinischen Bereich. (Aus den Best Practice Guidelines der Vereinigung der US-amerikanischen Blutbanken [33])

Blutprodukt	Indikation	Lagerung	Transportbedingungen	Maximale Lagerdauer
Erythrozytenkonzentrat	Erhöhung der Sauerstofftransportkapazität	1–6 °C	1–10 °C	ACD/CPD/CP2D: 21 Tage, CPDA-1: 35 Tage, Additivlösung: 42 Tage
Plasma	Korrektur einer Gerinnungsstörung, stellt die Endothelintegrität wieder her	Nach dem Auftauen: 1–6 °C	1–10 °C	Nach dem Auftauen: 5 Tage
Thrombozyten	Förderung der Hämostase	Konventionell: 20–24 °C mit kontinuierlicher, Agitation	Konventionell: 20–24 °C, maximale Zeit ohne Agitation: 30 h	Konventionell: 24 h bis 5 Tage, je nach Sammelsystem
		Kühl gelagert: 1–6 °C, Agitation optional	Kühl gelagert: 1–10 °C	Kühl gelagert: Anweisungen des Herstellers
Niedrig-titriges Vollblut Gruppe 0 (LTOWB)	Enthält alle Blutbestandteile	1–6 °C	1–10 °C	CPD: 21 Tage CPDA-1: 35 Tage

In Deutschland wird Vollblut derzeit nicht produziert oder verwendet.

#### Infobox 1 Vorteile der Vollblutgabe bei Traumapatienten im hämorrhagischen Schock. (Aus den Best Practice Guidelines der Vereinigung der US-amerikanischen Blutbanken [44])

- Vollblut vereinfacht die Transfusionslogistik durch ausgewogene Bereitstellung aller Blutbestandteile in einem Produkt
- Konzentrierteres Produkt im Vergleich zur Rekonstitution von Vollblut mit herkömmlichen Komponenten
- Liefert gekühlt gelagerte Thrombozyten, die in vitro und möglicherweise in vivo eine verbesserte hämostatische Funktion im Vergleich zu bei Raumtemperatur gelagerten Blutplättchen aufweisen
- Sorgt für eine längere Haltbarkeit der gelagerten Thrombozyten im Vergleich zur Lagerung bei Raumtemperatur
- Sorgt für die Verfügbarkeit von Blutplättchen, wo sie sonst vielleicht nicht verfügbar gewesen wären
- Reduziert die bakterielle Kontaminationsrate eines plättchenhaltigen Produkts
- Reduziert die Inzidenz von AB0-Fehltransfusionen
- Reduziert die Exposition der Spender
- Kann unter gleichen Bedingungen wie EK transportiert werden

### Patientensicherheit

Bluttransfusionen bergen Risiken wie Transfusionsreaktionen, Infektionen, Hypokalzämie und Unterkühlung. Ein prophylaktischer Einsatz von Blutprodukten ist daher nicht gerechtfertigt. Eine sichere und effiziente Nutzung der knappen Ressource Blut erfordert Richtlinien für die Anwendung mit definierten Transfusionstriggern. Für den präklinischen Einsatz ist es allerdings, wie oben angeführt, nicht einfach, solche Auslöser zu definieren. Existierende Scores basieren in der Regel auf innerklinisch erhobenen Daten und sind nicht prospektiv validiert, weshalb weitere „dynamische“ Parameter wie Blutungsrate, Blutungskontrolle, Zeit bis zur technischen Rettung und die Zeit bis zum Erreichen des nächstgelegenen Traumazentrums berücksichtigt werden sollten.

Allerdings sind Transfusionsreaktionen bei prähospitalen Bluttransfusionen selten. Ein systematisches Review zeigt eine Nebenwirkungsrate von nur 1 % [68]. Das ist nachvollziehbar, da das Risiko schwerer Reaktionen, wie AB0-Inkompatibilität, sinkt, wenn für nichttypisierte Patienten die Blutgruppe 0 verwendet wird. Zudem könnte ein schweres Trauma die immunologische Reaktion durch temporäre Immunsuppression abschwächen [69]. Leichtere Transfusionsreaktionen könnten, während der Polytraumabehandlung oder bei massiven Blutungen unerkannt bleiben. Die PAMPer-Studie, die umfangreichste prospektive Untersuchung zu prähospitalen Blutprodukten, berichtete über 2,2 % leichter transfusionsassoziierter Nebenwirkungen nach Frischplasmagabe [18]. Ein weiteres systematisches Review bestätigt die Sicherheit prähospitaler Bluttransfusionen, wobei hauptsächlich geringfügige Transfusionsreaktionen konstatiert wurden [70]. Zudem belegen zwei neuere randomisierte Studien keine erhöhten transfusionsassoziierten Komplikationen bei prähospital gegenüber bei Aufnahme transfundierten Patienten [71, 72].

### Logistik

Selleng et al. [73] haben die Risikoanalyse, Validierung und Logistik für ein prähospitales Transfusionsprogramm (EK, FFP und lyophilisiertes Plasma) gemäß den deutschen Rahmenbedingungen detailliert dargestellt. Nach sorgfältiger Validierung und unter Berücksichtigung sämtlicher Vorgaben der rechtlichen Anforderungen an eine prähospitale Transfusion (Erstellung einer SOP, Aufrechterhaltung der Kühlkette, Durchführung von Bedside-Tests etc.), ließen sich die Prozesse nahtlos in den Alltag der Blutbank und der Luftrettung einbinden. Durch die enge Zusammenarbeit von Blutbank, Rettungsdienst und beteiligten Krankenhäusern konnten nichtverwendete Blutprodukte ohne Qualitätsverlust in den Bestand der Blutbank zurückgeführt und erneut eingesetzt werden.

### Evidenz – prospektive Studien aus der Präklinik

Im Rahmen der Literatursuche haben wir 6 prospektive Studien gefunden, die das Patienten-Outcome bei Gabe von prähospitalen Blutprodukten untersuchten:

- Die randomisierte **COMBAT-Studie** [74] hat den Effekt von 2 Einheiten tiefgefrorenem FFP vs. tiefgefrorener Kochsalzlösung im innerstädtischen Bereich von Denver, USA, untersucht. Beides wurde am Einsatzort vor Gabe in einem Mikrowellenofen aufgetaut. Es wurden 144 Traumapatienten von 150 geplanten Patienten mit Zeichen eines hämorrhagischen Schocks eingeschlossen. Signifikante Unterschiede in der 28-Tage-Mortalität fanden sich nicht. (15 % Plasma vs. 10 % Kontrolle; Odds Ratio 1,54; 95 %-KI 0,60–3,98; *p* = 0,37). Da sich in der Zwischenanalyse kein Mortalitätsunterschied abzeichnete, wurde die Studie vorzeitig abgebrochen.
- Die **PAMPer-Studie **[18] war eine US-amerikanische, multizentrische, Cluster-randomisierte Studie, die Effekt und Sicherheit von 2 Einheiten FFP im Vergleich zur Standardversorgung bei 501 Traumapatienten mit Schockzeichen untersuchte. Die Studie randomisierte 27 Hubschrauberbasen für prähospitales Plasma oder Standardbehandlung. Das primäre Ergebnis war die 30-Tage-Mortalität, die in der Plasmagruppe im Vergleich zur Standardversorgungsgruppe um 10 % niedriger war (23 % gegenüber 33 %, 95 %-KI [(−18,6)–(−1,0); *p* = 0,03]). Die scheinbar widersprüchlichen Ergebnisse beider Studien wurden in zwei Metaanalysen beider Datensätze weiter differenziert, mit dem Ergebnis, dass sich im Studienarm ein Überlebensvorteil für Patienten mit stumpfem Trauma (OR 0,68; 95 %-KI [0,47–0,96; 0,03]) [75] sowie bei Patienten mit Transportzeiten über 20 min nachweisen ließ (OR 0,78; 95 %-KI [0,40–1,51; *p* = 0,46]) [76]. Bemerkenswert ist, dass in beiden Studien die Verletzungsschwere (PAMPer: ISS = 22, Combat: NISS = 27) nicht besonders hoch war. Die PAMPer-Studie hat mehrere sekundäre Datenanalysen nach sich gezogen, die auf spezielle Patientengruppen fokussieren. So scheint die prähospitale Gabe von Frischplasma einen günstigen Effekt auf die Mortalität bei Patienten mit schwerem Schädel-Hirn-Trauma mit einem GCS < 8 (166 Patienten eingeschlossen) zu haben. Die Autoren fanden eine mehr als 50 %ige Reduktion der 30-Tage-Mortalität bei Patienten, die von der Einsatzstelle direkt in die teilnehmenden Traumazentren transportiert wurden (OR 0,45; 95 %-KI, 0,26–0,80; *P* = 0,005) [77]. Eine weitere sekundäre Analyse der PAMPer-Studie [78] hat den Effekt der Transfusionsbehandlung an 407 initial hypotonen Patienten untersucht: Die Standardbehandlung bestand je nach Studienort aus isotonischer Kochsalzlösung (NaCl) oder aus isotonischer Kochsalzlösung plus EK. Dies führte zu 4 prähospitalen Transfusionsregimen: EK + Plasma, Plasma, EK und nur NaCl. Cox-Regression wurde benutzt, um den Zusammenhang zwischen den Gruppen und der risikoadjustierten 30-Tage-Mortalität zu bestimmen. Hier zeigte EK + Plasma den größten Effekt (OR 0,38; 95 %-KI 0,26–0,55, *p* < 0,001), gefolgt von Plasma (OR 0,57; 95 %-KI 0,36–0,91, *p* = 0,017) und EK (OR 0,68; 95 %-KI 0,49–0,95, *p* = 0,025) im Vergleich zu nur NaCl. Die Autoren schlussfolgern, dass Traumapatienten im hämorrhagischem Schock prähospitale Blutprodukte erhalten sollten, vorzugsweise EK + Plasma.
- Die **RePHILL-Studie** aus Großbritannien [71] hat erstmalig prospektiv und randomisiert die prähospitale Gabe von EK plus gefriergetrocknetem Plasma (Interventionsarm) mit dem Einsatz isotonischer Kochsalzlösung (Kontrollarm) bei Traumapatienten mit vermutetem hämorrhagischen Schock verglichen. Interessanterweise wurde als primäres Outcome ein kombinierter Endpunkt aus Episodenmortalität und Laktatakkumulation gewählt. Hier offenbarten sich bereits zwei erhebliche Designschwächen der Studie:
  1. Generell sollten die einzelnen Komponenten eines kombinierten Endpunkts in Schweregrad und klinischer Bedeutung ähnlich sein. Während eine Reduktion der Mortalität zweifelsohne klinisch relevant ist, ist die Lactat-Clearance jedoch weder ein harter Endpunkt noch ein validierter Outcome-Parameter bei Trauma. Daher ist die Laktatakkumulation für Dichotomisierung und binäre Interpretation ungeeignet.
  2. Der gewählte Stichprobenumfang basierte auf der Annahme, dass die Intervention die Inzidenz des kombinierten Endpunkts von 20 % im Kontrollarm auf 10 % im Studienarm reduzieren würde. Eine 50 %ige Risikoreduktion bei Ergebnissen wie Mortalität, die vielen Störvariablen unterliegt, erscheint jedoch kaum plausibel.
  Die Inzidenz des kombinierten Endpunkts war in beiden Gruppen nahezu gleich: Im Interventionsarm traten bei 128 von 199 Patienten (64 %) Ereignisse auf, verglichen mit 136 von 210 Patienten (65 %) in der Kontrollgruppe (RR 1,01; 95 %-KI 0,88–1,17; *p* = 0,86).
  Die Inzidenz des kombinierten Endpunkts war in beiden Gruppen nahezu gleich: Im Interventionsarm traten bei 128 von 199 Patienten (64 %) Ereignisse auf, verglichen mit 136 von 210 Patienten (65 %) in der Kontrollgruppe (RR 1,01; 95 %-KI 0,88–1,17; *p* = 0,86). Als sekundäres Ergebnis wurde eine 7 %ige Reduktion der 3-Stunden-Mortalität festgestellt: Im Interventionsarm verstarben 32 von 197 Patienten (16 %), während es im Kontrollarm 46 von 208 Patienten (22 %) waren (RR 0,75; 95 %-KI 0,50–1,13; *p* = 0,17).
  Ein weiteres sekundäres Ergebnis war eine 4 %ige Reduktion der 30-Tage-Mortalität, die in der Kontrollgruppe bei 45 % lag und in der Interventionsgruppe auf 42 % sank (RR 0,94; 95 %-KI 0,76–1,17; *p* = 0,59). Keines dieser Ergebnisse erreichte statistische Signifikanz, und die Autoren schlussfolgerten, dass die Gabe von EK und Plasma keinen Vorteil gegenüber isotonischer Kochsalzlösung bietet.
  Dennoch zeichnet sich im Interventionsarm in allen untersuchten Parametern ein positiver Trend ab, der klinisch durchaus relevant sein könnte. Bedauerlicherweise musste die RePHILL-Studie wegen erheblicher Rekrutierungsprobleme vorzeitig abgebrochen werden und konnte statt, wie geplant, 490 Patienten lediglich 432 Patienten rekrutieren. Zusammenfassend kann festgestellt werden, dass RePHILL, bedingt durch die bereits angeführten Designschwächen und die Rekrutierungsprobleme, nicht die statistische Power hatte, um eine weniger als 50 %ige Mortalitätsreduktion zu detektieren, was eine unrealistische Annahme war. Leider versäumten die Autoren es auch, eine mortalitätsbezogene Subgruppenanalyse durchzuführen. Diese Faktoren schränken die Aussagekraft der Studie erheblich ein [79].
- Die französische **PREHO-PLYO-Studie** [72] war eine randomisierte kontrollierte Studie, die bei schwer verletzten Patienten den Effekt von prähospital verabreichtem, lyophilisiertem Plasma vs. isotonischer Kochsalzlösung auf die Gerinnung untersucht hat. Insgesamt wurden 134 Patienten eingeschlossen, davon 68 im Studienarm und 66 in der Kontrollgruppe. Mediane INR-Werte waren 1,21 (IQR 1,12–1,49) im Studienarm und 1,20 (IQR 1,10–1,39) in der Kontrollgruppe. (Median Differenz −0,01 [IQR −0,09 to 0,08]; *p* = 0,88). Die Gruppen unterschieden sich nicht im Bedarf einer Massivtransfusion (7 [10,3 %] vs. 4 [6,1 %]; OR 1,78 [95 %-KI, 0,42–8,68]; *p* = 0,37) oder der 30-Tage-Mortalität (OR 1,07 [95 %-KI, 0,44–2,61]; *p* = 0,89). Die Autoren konkludieren, dass die prähospitale Gabe von lyophilisiertem Plasma keinen nachweisbaren Effekt auf die Gerinnungsparameter bei schwerverletzten Patienten hat. Die geringen Mortalitätsunterschiede zwischen den Gruppen sind jedoch in Anbetracht der niedrigen Fallzahlen nicht aussagekräftig.
- Die randomisierte **FIinTIC-Studie** [80] untersuchte die prähospitale Gabe von Fibrinogenkonzentrat an 53 Traumapatienten im hämorrhagischen Schock vs. Placebo. Eine frühzeitige Verabreichung von Fibrinogenkonzentrat in der Traumaversorgung stabilisierte die Fibrinogenwerte bei Aufnahme und verbessert die Blutgerinnung laborchemisch. Ob dies zu weniger Transfusionen oder Komplikationen bei Traumapatienten mit Blutungsrisiko führt, war aufgrund der niedrigen Fallzahlen nicht zu klären.
- Eine aktuelle prospektive Kohortenstudie (*n* = 909) aus Großbritannien [81] hat den Effekt von EK plus Plasma gegen EK (retrospektive Kontrollgruppe, *n* = 223) bei Schwerverletzten untersucht. Der Studienarm bestand aus 2 Gruppen: eine Gruppe erhielt mit Frischplasma rekonstituiertes EK (EKP-Gruppe, *n* = 295) und die andere erhielt EK plus Frischplasma (EK + P, *n* = 395). Die Gabe von EKP und EK + P war mit niedrigerer 24-h-Mortalität assoziiert als die alleinige Gabe von EK. (OR EK + P 0,69, 95%-KI: 0,52–0,92), (OR EKP 0,60, 95%-KI: 0,32–1,13). Die 30-Tage-Mortalität jedoch, war in allen 3 Gruppen gleich. Als Besonderheit stellten sich für das penetrierende Trauma die Ergebnisse etwas besser dar (EK + P: aOR 0,22 (95%-KI: 0,10–0,53); EKP: aOR 0,39 (95%-KI: 0,20–0,76)).

### Nationale Perspektive – Umsetzung im deutschen Rettungsdienst

Nach vorsichtiger Einschätzung erscheint die prähospitale Gabe von Blutprodukten nicht nur im militärischen Bereich sinnvoll. In vielen Ländern ist sie bereits fest etablierter Bestandteil der Rettungskette. Empfehlungen zur Implementierung, wie z. B. aus Maryland, USA, könnten als Orientierungshilfe dienen [82]. Die Identifikationen der Patientengruppen, die am ehesten profitieren würden, sowie der geeigneten Blutprodukte stehen allerdings noch aus und werden in Deutschland kontrovers diskutiert.

Die flächendeckende Vorhaltung im Rettungsdienst bedarf abgestimmter Konzepte, die regionale Bedarfe bei möglichst überregionaler Versorgungssicherheit berücksichtigt. Die lokale Umsetzung an einzelnen Standorten, z. B. im Bereich der Luftrettung, zeigt, dass dies bei Anbindung an eine lokale Blutbank relativ problemlos möglich ist.

Allerdings könnte auch der Einsatz von Drohnen, die direkt von transfusionsmedizinischen Einrichtungen oder Blutbanken aus starten könnten, exploriert werden.

In Deutschland beträgt die Inzidenz des traumabedingten Kreislaufschocks 38/100.000 Einwohnern pro Jahr [83]. In aktuellen retrospektiven Untersuchungen wird von 300–1800 [84, 85] Patienten jährlich ausgegangen, die von einer prähospitalen Transfusion profitieren könnten. Die praktische Erfahrung an transfundierenden deutschen Luftrettungsstandorten zeigt eine Durchführungshäufigkeit von etwa einer Transfusion pro Monat, mit saisonalen Schwankungen (eigene Zahlen, in Publikation).

Grundsätzlich ist bei der Vorhaltung prähospitaler Blutprodukte zu beachten, das neben der Transfusionsindikation Trauma, zumindest weltweit, die Indikation Nontrauma in ca. 50 % der Fälle eine relevante Größe darstellt [86].

Die im internationalen Vergleich extrem hohe Dichte notärztlicher Versorgung in Deutschland hat zu einer Verschiebung der Einsatzschwerpunkte der Rettungsdienste geführt. Die meisten Notarztsysteme haben daher nur noch geringe Erfahrung im Umgang mit schwerstverletzten Patienten. Dieser Umstand, gepaart mit einer stark fragmentierten, kommunalen Organisation der Rettungsdienste, fördert die vorherrschende Meinung, dass Bluttransfusionen im präklinischen Bereich nicht erforderlich seien [15]. Dadurch wurden die Diskussion und Entwicklung prähospitaler Bluttransfusionsprogramme in Deutschland maßgeblich verzögert.

Der wissenschaftlich nachweisbare Nutzen prähospitaler Blutprodukte bleibt umstritten, da umfassende prospektive Studien aufgrund der Komplexität der Fragestellung an der notwendigen Patientenzahl scheiterten. Prospektive Registerstudien sind wahrscheinlich am ehesten geeignet, die noch ausstehenden Fragen zu beantworten. Einen ersten Schritt in diese Richtung haben in enger Abstimmung die medizinisch Verantwortlichen der Luftrettungsbetreiber, ergänzt um einzelne bodengebundene Systeme, mit der Idee zur Gründung des „Blut im Notarztdienst-Register (BiNAR)“ unter dem Dach der BAND e. V. unternommen [87].

Ebenso könnten weitere vergleichende Outcomeuntersuchungen in Kooperation mit dem Trauma Register der DGU durchgeführt werden, um den Effekt von PHBT auf die gesamte Versorgungskette zu beleuchten.

### Schlussfolgerung

Obwohl die theoretische Grundlage für die prähospitale Blutgabe solide erscheint und ihre Anwendung in vielen europäischen Ländern bereits etabliert wurde, fehlt in Deutschland noch eine umfassende Bewertung des Nutzens im Vergleich zum Aufwand im zivilen Rettungsdienst. Die Erfahrungen aus den angeführten Studien legen nahe, dass die Beantwortung der offenen Fragen aufgrund der Komplexität und des damit verbundenen Aufwands durch randomisierte kontrollierte Studien (RCT) nicht erfolgen kann. Stattdessen scheinen Registerstudien eher geeignet zu sein, um diese Herausforderungen zu bewältigen. Deutschland ist mit dem Trauma Register der Deutschen Gesellschaft für Unfallchirurgie gut für die Auswertung im Rahmen solcher Studien aufgestellt, was eine einzigartige Gelegenheit böte, die Effekte prähospitaler Blutgaben auf einer breiten Datengrundlage zu untersuchen und somit die Versorgung von Traumapatienten gezielt zu verbessern.

## Fazit für die Praxis

- Eine prähospitale Bluttransfusion (PHBT) könnte zur Verbesserung des Outcomes schwer verletzter Patienten, insbesondere bei langen Transportwegen, beitragen.
- Identifikation der Patientengruppen, die von PHBT profitieren, ist entscheidend, um eine zielgerichtete und effiziente Nutzung dieser Maßnahme zu gewährleisten.
- Die hohe Dichte von Notarztstandorten und die fragmentierte Struktur des Rettungsdienstes in Deutschland führen zu einer niedrigen Traumaexposition pro System, was die flächendeckende Anwendung von PHBT erschwert.
- Prospektive Registerstudien in Kooperation mit dem Traumaregister der DGU sind am ehesten geeignet, um offene Fragen zu PHBT zu klären.
- Die Auswahl und Bereitstellung geeigneter Blutprodukte für PHBT erfordert eine fundierte Logistik und Koordination zwischen Rettungsdiensten und transfusionsmedizinischen Einrichtungen.
- Weiterbildung und Training von Notfallpersonal in der Handhabung von PHBT sind für eine sichere und effektive Anwendung erforderlich.
- Eine enge Zusammenarbeit zwischen Notfallmedizin, Transfusionsmedizin und Traumazentren ist für die Weiterentwicklung und optimale Integration von PHBT in die prähospitale Notfallversorgung notwendig.

## References

[1] Eastridge BJ, Holcomb JB, Shackelford S (2019) Outcomes of traumatic hemorrhagic shock and the epidemiology of preventable death from injury. Transfusion 59(S2):1423–1428. 10.1111/trf.15161 30980749 10.1111/trf.15161

[2] Kiel F (1966) Development of a blood program in Vietnam. Mil Med 131(12):1469–1482 4962998

[3] Rossaint R, Afshari A, Bouillon B, Cerny V, Cimpoesu D, Curry N, Duranteau J, Filipescu D, Grottke O, Grønlykke L, Harrois A, Hunt BJ, Kaserer A, Komadina R, Madsen MH, Maegele M, Mora L, Riddez L, Romero CS, Samama CM, Vincent JL, Wiberg S, Spahn DR (2023) The European guideline on management of major bleeding and coagulopathy following trauma: sixth edition. Crit Care 27(1):80. 10.1186/s13054-023-04327-7 36859355 10.1186/s13054-023-04327-7 PMC9977110

[4] Moran CG, Lecky F, Bouamra O, Lawrence T, Edwards A, Woodford M, Willett K, Coats TJ (2018) Changing the system—major trauma patients and their outcomes in the NHS (England) 2008–17. EClinicalMedicine 2–3:13–21. 10.1016/j.eclinm.2018.07.001 31193723 10.1016/j.eclinm.2018.07.001 PMC6537569

[5] Swerts F, Mathonet PY, Ghuysen A, D’Orio V, Minon JM, Tonglet M (2019) Early identification of trauma patients in need for emergent transfusion: results of a single-center retrospective study evaluating three scoring systems. Eur J Trauma Emerg Surg 45(4):681–686. 10.1007/s00068-018-0965-0 29855669 10.1007/s00068-018-0965-0

[6] Dudaryk R, Heim C, Ruetzler K, Pivalizza EG (2022) Pro-con debate: prehospital blood transfusion-should it be adopted for civilian trauma? Anesth Analg 134(4):678–682. 10.1213/ANE.0000000000005747 35299208 10.1213/ANE.0000000000005747

[7] Robertson LB (1916) The transfusion of whole blood: a suggestion for its more frequent employment in war surgery. Br Med J 2(2897):38–40. 10.1136/bmj.2.2897.38 20768200 10.1136/bmj.2.2897.38 PMC2348759

[8] Cannon WB (1923) Traumatic shock. D. Appleton and Company, New York

[9] Robertson OH (1918) Transfusion WITH preserved red blood cells. Br Med J 1(2999):691–695. 10.1136/bmj.1.2999.691 20769077 10.1136/bmj.1.2999.691 PMC2340627

[10] Edwards FR, Kay J, Davie TB (1940) Preparation and use of dried plasma for transfusion. Br Med J 1(4131):377–381. 10.1136/bmj.1.4131.377 20782987 10.1136/bmj.1.4131.377 PMC2176676

[11] Kendrick DB (1964) Blood program in world war II. Office of the Surgeon General, Washington, D.C.

[12] Negovsky V (1946) Treatment of agonal and clinical death. Nature 157:163. 10.1038/157163a0 21065852 10.1038/157163a0

[13] Kotwal RS, Scott LLF, Janak JC, Tarpey BW, Howard JT, Mazuchowski EL, Butler FK, Shackelford SA, Gurney JM, Stockinger ZT (2018) The effect of prehospital transport time, injury severity, and blood transfusion on survival of US military casualties in Iraq. J Trauma Acute Care Surg 85(1):S112–S121. 10.1097/TA.0000000000001798 29334570 10.1097/TA.0000000000001798

[14] Eastridge BJ, Mabry RL, Seguin P, Cantrell J, Tops T, Uribe P, Mallett O, Zubko T, Oetjen-Gerdes L, Rasmussen TE, Butler FK, Kotwal RS, Holcomb JB, Wade C, Champion H, Lawnick M, Moores L, Blackbourne LH (2012) Death on the battlefield (2001–2011): implications for the future of combat casualty care. J Trauma Acute Care Surg 73(6):S431–7. 10.1097/TA.0b013e3182755dcc 23192066 10.1097/TA.0b013e3182755dcc

[15] Thies KC, Truhlář A, Keene D, Hinkelbein J, Rützler K, Brazzi L, Vivien B (2020) Pre-hospital blood transfusion—an ESA survey of European practice. Scand J Trauma Resusc Emerg Med 28(1):79. 10.1186/s13049-020-00774-1 32795320 10.1186/s13049-020-00774-1 PMC7427720

[16] Kleber C, Giesecke MT, Tsokos M, Haas NP, Buschmann CT (2013) Trauma-related preventable deaths in Berlin 2010: need to change prehospital management strategies and trauma management education. World J Surg 37(5):1154–1161. 10.1007/s00268-013-1964-2 23430005 10.1007/s00268-013-1964-2

[17] Cannon JW (2018) Hemorrhagic shock. N Engl J Med 378(4):370–379. 10.1056/NEJMra1705649 29365303 10.1056/NEJMra1705649

[18] Cotton BA, Guy JS, Morris JA Jr, Abumrad NN (2006) The cellular, metabolic, and systemic consequences of aggressive fluid resuscitation strategies. Shock 26(2):115–121. 10.1097/01.shk.0000209564.84822.f2 16878017 10.1097/01.shk.0000209564.84822.f2

[19] Brinck T, Handolin L, Lefering R (2016) The effect of evolving fluid resuscitation on the outcome of severely injured patients: an 8‑year experience at a tertiary trauma. Scand J Surg 105(2):109–116 25989810 10.1177/1457496915586650

[20] Dutton RP (2012) Haemostatic resuscitation. Br J Anaesth 109(1):i39–i46. 10.1093/bja/aes389 23242750 10.1093/bja/aes389

[21] Leibner E, Andreae M, Galvagno SM, Scalea T (2020) Damage control resuscitation. Clin Exp Emerg Med 7(1):5–13. 10.15441/ceem.19.089 32252128 10.15441/ceem.19.089 PMC7141982

[22] Shackelford SA, del Junco DJ, Powell-Dunford N et al (2017) Association of prehospital blood product transfusion during medical evacuation of combat casualties in Afghanistan with acute and 30-day survival. JAMA 318(16):1581–1591. 10.1001/jama.2017.15097 29067429 10.1001/jama.2017.15097 PMC5818807

[23] Meyer DE, Vincent LE, Fox EE, O’Keeffe T, Inaba K, Bulger E et al (2017) Every minute counts: time to delivery of initial massive transfusion cooler and its impact on mortality. J Trauma Acute Care Surg 83(1):19–24 28452870 10.1097/TA.0000000000001531 PMC5526458

[24] Powell EK, Hinckley WR, Gottula A, Hart KW, Lindsell CJ, McMullan JT (2016) Shorter times to packed red blood cell transfusion are associated with decreased risk of death in traumatically injured patients. J Trauma Acute Care Surg 81(3):458–462. 10.1097/TA.0000000000001078 27050884 10.1097/TA.0000000000001078

[25] AWMF online (2022) S3 Leitlinie Polytrauma/Schwerverletztenversorgung. https://register.awmf.org/de/leitlinien/detail/187-023

[26] Bichot A, Pasquier P, Martinaud C, Corcostegui SP, Boutot F, Cazes N, Boutillier du Retail C, Travers S, Galant J (2023) Use of prehospital transfusion by French emergency medical services: a national survey. Transfusion 63(3):S241–S248. 10.1111/trf.17374 37071770 10.1111/trf.17374

[27] Naumann DN, Hancox JM, Raitt J, Smith IM, Crombie N, Doughty H, Perkins GD, Midwinter MJ, RESCUER Collaborators (2018) What fluids are given during air ambulance treatment of patients with trauma in the UK, and what might this mean for the future? Results from the RESCUER observational cohort study. BMJ Open 8(1):e19627. 10.1136/bmjopen-2017-019627 29362272 10.1136/bmjopen-2017-019627 PMC5786144

[28] Mercer J (2022) 774 survey to investigate the current use of pre-hospital blood product by air ambulance services in the United Kingdom. Emerg Med J 39:257. 10.1136/emermed-2022-RCEM.29

[29] Dalton AM (1993) Use of blood transfusions by helicopter emergency medical services: is it safe? Injury 24(8):509–510. 10.1016/0020-1383(93)90023-y 8244539 10.1016/0020-1383(93)90023-y

[30] Greene A, Trojanowski J, Shih AW, Evans R, Chang E, Nahirniak S, Pearson D, Prokopchuk-Gauk O, Martin D, Musuka C, Seidl C, Peddle M, Lin Y, Smith JA, MacDonald S, Richards L, Farrell M, Nolan B (2023) A descriptive analysis of the Canadian prehospital and transport transfusion (CAN-PATT) network. Resusc Plus 13:100357. 10.1016/j.resplu.2022.100357 36691447 10.1016/j.resplu.2022.100357 PMC9860513

[31] Reid C (2013) Verfahrensanweisung für den Luftrettungsdienst. https://nswhems.files.wordpress.com/2015/12/heli-cli-11-blood-management-v2.pdf

[32] Voller J US armed forces joint trauma system clinical practice guideline on prehospital blood transfusion 10.55460/P685-L7R7 34969121

[33] THOR 2021. https://www.tandfonline.com/doi/full/10.1080/10903127.2021.1995089

[34] Hooper TJ, De Pasquale M, Strandenes G, Sunde G, Ward KR (2014) Challenges and possibilities in forward resuscitation. Shock 41(1):13–20. 10.1097/SHK.0000000000000096 24296432 10.1097/SHK.0000000000000096

[35] O’Reilly DJ, Morrison JJ, Jansen JO, Nordmann G, Rasmussen TE, Midwinter MJ, Doughty H (2014) Initial UK experience of prehospital blood transfusion in combat casualties. J Trauma Acute Care Surg 77(3):S66–70. 10.1097/TA.0000000000000342 25159364 10.1097/TA.0000000000000342

[36] Penn-Barwell JG, Roberts SA, Midwinter MJ, Bishop JR (2015) Improved survival in UK combat casualties from Iraq and Afghanistan: 2003–2012. J Trauma Acute Care Surg 78(5):1014–1020. 10.1097/TA.0000000000000580 25909424 10.1097/TA.0000000000000580

[37] Yin G, Radulovic N, O’Neill M, Lightfoot D, Nolan B (2023) Predictors of transfusion in trauma and their utility in the prehospital environment: a scoping review. Prehosp Emerg Care 27(5):575–585. 10.1080/10903127.2022.2120935 36066217 10.1080/10903127.2022.2120935

[38] van Turenhout EC, Bossers SM, Loer SA, Giannakopoulos GF, Schwarte LA, Schober P (2020) Pre-hospital transfusion of red blood cells. Part 1: a scoping review of current practice and transfusion triggers. Transfus Med 30(2):86–105. 10.1111/tme.12667 32080942 10.1111/tme.12667 PMC7317877

[39] Meyer DE et al (2018) A comparison of resuscitation intensity and critical administration threshold in predicting early mortality among bleeding patients: a multicenter validation in 680 major transfusion patients. J Trauma Acute Care Surg 85(4):691–696. 10.1097/TA.0000000000002020 29985236 10.1097/TA.0000000000002020 PMC6158088

[40] Maegele M, Lefering R, Wafaisade A, Theodorou P, Wutzler S, Fischer P, Bouillon B, Paffrath T, Trauma Registry of Deutsche Gesellschaft für Unfallchirurgie (2011) Revalidation and update of the TASH-Score: a scoring system to predict the probability for massive transfusion as a surrogate for life-threatening haemorrhage after severe injury. Vox Sang 100(2):231–238. 10.1111/j.1423-0410.2010.01387.x 20735809 10.1111/j.1423-0410.2010.01387.x

[41] Yücel N, Lefering R, Maegele M, Vorweg M, Tjardes T, Ruchholtz S, Neugebauer EA, Wappler F, Bouillon B, Rixen D, Polytrauma Study Group of the German Trauma Society (2006) Trauma associated severe hemorrhage (TASH)-score: probability of mass transfusion as surrogate for life threatening hemorrhage after multiple trauma. J Trauma 60(6):1228–1236. 10.1097/01.ta.0000220386.84012.bf (discussion 1236–7) 16766965 10.1097/01.ta.0000220386.84012.bf

[42] Horst K, Lentzen R, Tonglet M, Mert Ü, Lichte P, Weber CD, Kobbe P, Heussen N, Hildebrand F (2020) Validation of the mTICCS score as a useful tool for the early prediction of a massive transfusion in patients with a traumatic hemorrhage. J Clin Med 9(4):945. 10.3390/jcm9040945 32235488 10.3390/jcm9040945 PMC7230969

[43] Horst K, Lichte P, Bläsius F, Weber CD, Tonglet M, Kobbe P, Heussen N, Hildebrand F (2022) mTICCS and its inter-rater reliability to predict the need for massive transfusion in severely injured patients. Eur J Trauma Emerg Surg 48(1):367–372. 10.1007/s00068-020-01523-w 33051727 10.1007/s00068-020-01523-w PMC8825405

[44] Tonglet M, Lefering R, Minon JM, Ghuysen A, D’Orio V, Hildebrand F, Pape HC, Horst K (2017) Prehospital identification of trauma patients requiring transfusion: results of a retrospective study evaluating the use of the trauma induced coagulopathy clinical score (TICCS) in 33,385 patients from the TraumaRegister DGU®. Acta Chir Belg 117(6):385–390. 10.1080/00015458.2017.1341148 28639537 10.1080/00015458.2017.1341148

[45] Tonglet ML, Minon JM, Seidel L, Poplavsky JL, Vergnion M (2014) Prehospital identification of trauma patients with early acute coagulopathy and massive bleeding: results of a prospective non-interventional clinical trial evaluating the trauma induced coagulopathy clinical score (TICCS). Crit Care 18(6):648. 10.1186/s13054-014-0648-0 25425230 10.1186/s13054-014-0648-0 PMC4279963

[46] Gaessler H, Helm M, Kulla M et al (2023) Prehospital predictors of the need for transfusion in patients with major trauma. Eur J Trauma Emerg Surg 49:803–812. 10.1007/s00068-022-02132-5 36222858 10.1007/s00068-022-02132-5 PMC10175474

[47] Griggs JE, Lyon RM, Sherriff M, Barrett JW, Wareham G, ter Avest E (2022) Predictive clinical utility of pre-hospital point of care lactate for transfusion of blood product in patients with suspected traumatic haemorrhage: derivation of a decision-support tool. Scand J Trauma Resusc Emerg Med 30(1):72. 10.1186/s13049-022-01061-x 36514084 10.1186/s13049-022-01061-x PMC9749287

[48] Selleng K, Jenichen G, Denker K, Selleng S, Müllejans B, Greinacher A (2017) Emergency transfusion of patients with unknown blood type with blood group O Rhesus D positive red blood cell concentrates: a prospective, single-centre, observational study. Lancet Haematol 4(5):e218–e224. 10.1016/S2352-3026(17)30051-0 28389344 10.1016/S2352-3026(17)30051-0

[49] Selleng K, Greinacher A (2021) 10 years of experience with the first thawed plasma bank in Germany. Transfus Med Hemother 48(6):350–357. 10.1159/000519700 35082566 10.1159/000519700 PMC8739389

[50] de Roulet A, Kerby JD, Weinberg JA, Lewis RH Jr, Hudgins JP, Shulman IA, Fox EE, Holcomb JB, Brasel KJ, Bulger EM, Cohen MJ, Cotton BA, Fabian TC, O’Keeffe T, Rizoli S, Scalea TM, Schreiber MA, Inaba K, PROPPR Study Group (2020) Group A emergency-release plasma in trauma patients requiring massive transfusion. J Trauma Acute Care Surg 89(6):1061–1067. 10.1097/TA.0000000000002903 32890339 10.1097/TA.0000000000002903 PMC7830815

[51] Zur M, Glassberg E, Gorenbein P, Epstein E, Eisenkraft A, Misgav M, Avramovich E (2019) Freeze-dried plasma stability under prehospital field conditions. Transfusion 59:3485–3490. 10.1111/trf.15533 31568580 10.1111/trf.15533

[52] Garrigue D, Godier A, Glacet A, Labreuche J, Kipnis E, Paris C, Duhamel A, Resch E, Bauters A, Machuron F, Renom P, Goldstein P, Tavernier B, Sailliol A, Susen S (2018) French lyophilized plasma versus fresh frozen plasma for the initial management of trauma-induced coagulopathy: a randomized open-label trial. J Thromb Haemost 16(3):481–489. 10.1111/jth.13929 29274254 10.1111/jth.13929

[53] Pusateri AE, Malloy WW, Sauer D, Benov A, Corley JB, Rambharose S, Wallis L, Tiller MM, Cardin S, Glassberg E, Weiskopf RB (2022) Use of dried plasma in prehospital and austere environments. Anesthesiology 136(2):327–335. 10.1097/ALN.0000000000004089 34919639 10.1097/ALN.0000000000004089

[54] Moore EE, Moore HB, Kornblith LZ, Neal MD, Hoffman M, Mutch NJ, Schöchl H, Hunt BJ, Sauaia A (2021) Trauma-induced coagulopathy. Nat Rev Dis Primers 7(1):30. 10.1038/s41572-021-00264-3 33927200 10.1038/s41572-021-00264-3 PMC9107773

[55] McQuilten ZK, Wood EM, Bailey M, Cameron PA, Cooper DJ (2017) Fibrinogen is an independent predictor of mortality in major trauma patients: a five-year statewide cohort study. Injury 48(5):1074–1081. 10.1016/j.injury.2016.11.021 28190583 10.1016/j.injury.2016.11.021

[56] Itagaki Y, Hayakawa M, Maekawa K, Saito T, Kodate A, Honma Y, Mizugaki A, Yoshida T, Ohyasu T, Katabami K, Wada T (2020) Early administration of fibrinogen concentrate is associated with improved survival among severe trauma patients: a single-centre propensity score-matched analysis. World J Emerg Surg 15:7. 10.1186/s13017-020-0291-9 31956337 10.1186/s13017-020-0291-9 PMC6961302

[57] Empfehlung der ‚Agency for Clinical Innovation‘ der Gesundheitsbehörde NSW. https://aci.health.nsw.gov.au/__data/assets/pdf_file/0004/716566/ACI-Fibrinogen-administration-in-prehospital-trauma.pdf

[58] Brill JB, Mueck KM, Tang B, Sandoval M, Cotton ME, Cameron McCoy C, Cotton BA (2023) Is low-titer group O whole blood truly a universal blood product? J Am Coll Surg 236(3):506–513. 10.1097/XCS.0000000000000489 36730210 10.1097/XCS.0000000000000489

[59] Brill JB, Tang B, Hatton G, Mueck KM, McCoy CC, Kao LS, Cotton BA (2022) Impact of incorporating whole blood into hemorrhagic shock resuscitation: analysis of 1,377 consecutive trauma patients receiving emergency-release uncrossmatched blood products. J Am Coll Surg 234(4):408–418. 10.1097/XCS.0000000000000086 35290259 10.1097/XCS.0000000000000086

[60] McCoy CC, Montgomery K, Cotton ME, Meyer DE, Wade CE, Cotton BA (2021) Can RH+ whole blood be safely used as an alternative to RH-product? An analysis of efforts to improve the sustainability of a hospital’s low titer group O whole blood program. J Trauma Acute Care Surg 91(4):627–633. 10.1097/TA.0000000000003342 34238860 10.1097/TA.0000000000003342

[61] Cotton BA, Guy JS, Morris JA Jr, Abumrad NN (2006) The cellular, metabolic, and systemic consequences of aggressive fluid resuscitation strategies. Shock 26(2):115–121. 10.1097/01.shk.0000209564.84822.f2 16878017 10.1097/01.shk.0000209564.84822.f2

[62] Torres CM, Kent A, Scantling D, Joseph B, Haut ER, Sakran JV (2023) Association of whole blood with survival among patients presenting with severe hemorrhage in US and Canadian adult civilian trauma centers. JAMA Surg 158(5):532–540. 10.1001/jamasurg.2022.6978 36652255 10.1001/jamasurg.2022.6978 PMC9857728

[63] Yazer MH, Spinella PC, Anto V, Dunbar NM (2021) Survey of group A plasma and low-titer group O whole blood use in trauma resuscitation at adult civilian level 1 trauma centers in the US. Transfusion 61(6):1757–1763. 10.1111/trf.16394 33797100 10.1111/trf.16394

[64] Martinaud C, Tiberghien P, Bégué S, Sailliol A, Gross S, Pouget T, Ausset S (2019) Rational and design of the T‑STORHM study: a prospective randomized trial comparing fresh whole blood to blood components for acutely bleeding trauma patients. Transfus Clin Biol 26(4):198–201. 10.1016/j.tracli.2019.09.004 31645305 10.1016/j.tracli.2019.09.004

[65] NHS Blood and Transplant Clinical Trials Unit Study of whole blood in frontline trauma 10.1186/ISRCTN23657907

[66] Smith JE, Barnard EBG, Brown-O’Sullivan C, Cardigan R, Davies J, Hawton A, Laing E, Lucas J, Lyon R, Perkins GD, Smith L, Stanworth SJ, Weaver A, Woolley T, Green L (2023) The SWiFT trial (study of whole blood in frontline trauma)—the clinical and cost effectiveness of pre-hospital whole blood versus standard care in patients with life-threatening traumatic haemorrhage: study protocol for a multi-centre randomised controlled trial. Trials 24(1):725. 10.1186/s13063-023-07711-4 37964393 10.1186/s13063-023-07711-4 PMC10644622

[67] Sunde GA, Bjerkvig C, Bekkevold M, Kristoffersen EK, Strandenes G, Bruserud Ø, Apelseth TO, Heltne JK (2022) Implementation of a low-titre whole blood transfusion program in a civilian helicopter emergency medical service. Scand J Trauma Resusc Emerg Med 30(1):65. 10.1186/s13049-022-01051-z 36494743 10.1186/s13049-022-01051-z PMC9733220

[68] Rijnhout TWH, Wever KE, Marinus RHAR, Hoogerwerf N, Geeraedts LMG Jr, Tan ECTH (2019) Is prehospital blood transfusion effective and safe in haemorrhagic trauma patients? A systematic review and meta-analysis. Injury 50(5):1017–1027. 10.1016/j.injury.2019.03.033 30928164 10.1016/j.injury.2019.03.033

[69] Islam MN, Bradley BA, Ceredig R (2016) Sterile post-traumatic immunosuppression. Clin Transl Immunology 5(4):e77. 10.1038/cti.2016.13 27195120 10.1038/cti.2016.13 PMC4855263

[70] Shand S, Curtis K, Dinh M, Burns B (2019) What is the impact of prehospital blood product administration for patients with catastrophic haemorrhage: an integrative review. Injury 50(2):226–234. 10.1016/j.injury.2018.11.049 30578085 10.1016/j.injury.2018.11.049

[71] Crombie N, Doughty HA, Bishop JRB, Desai A, Dixon EF, Hancox JM, Herbert MJ, Leech C, Lewis SJ, Nash MR, Naumann DN, Slinn G, Smith H, Smith IM, Wale RK, Wilson A, Ives N, Perkins GD, RePHILL collaborative group (2022) Resuscitation with blood products in patients with trauma-related haemorrhagic shock receiving prehospital care (RePHILL): a multicentre, open-label, randomised, controlled, phase 3 trial. Lancet Haematol 9(4):e250–e261. 10.1016/S2352-3026(22)00040-0 35271808 10.1016/S2352-3026(22)00040-0 PMC8960285

[72] Jost D, Lemoine S, Lemoine F, Derkenne C, Beaume S, Lanoë V, Maurin O, Louis-Delaurière E, Delacote M, Dang-Minh P, Franchin-Frattini M, Bihannic R, Savary D, Levrat A, Baudouin C, Trichereau J, Salomé M, Frattini B, Ha VHT, Jouffroy R, Seguineau E, Titreville R, Roquet F, Stibbe O, Vivien B, Verret C, Bignand M, Travers S, Martinaud C, Arock M, Raux M, Prunet B, Ausset S, Sailliol A, Tourtier JP (2022) Prehospital lyophilized plasma transfusion for trauma-induced coagulopathy in patients at risk for hemorrhagic shock: a randomized clinical trial. JAMA Netw Open 5(7):e2223619. 10.1001/jamanetworkopen.2022.23619 35881397 10.1001/jamanetworkopen.2022.23619 PMC9327575

[73] Selleng K, Baschin M, Henkel B, Jenichen G, Thies KC, Rudolph M, Reifferscheid F, Braun J, Hannich M, Winter T, Hahnenkamp K, Greinacher A (2021) Blood product supply for a helicopter emergency medical service. Transfus Med Hemother 48(6):332–341. 10.1159/000519825 35082564 10.1159/000519825 PMC8740152

[74] Moore HB, Moore EE, Chapman MP, McVaney K, Bryskiewicz G, Blechar R, Chin T, Burlew CC, Pieracci F, West FB, Fleming CD, Ghasabyan A, Chandler J, Silliman CC, Banerjee A, Sauaia A (2018) Plasma-first resuscitation to treat haemorrhagic shock during emergency ground transportation in an urban area: a randomised trial. Lancet 392(10144):283–291. 10.1016/S0140-6736(18)31553-8 30032977 10.1016/S0140-6736(18)31553-8 PMC6284829

[75] Reitz KM, Moore HB, Guyette FX, Sauaia A, Pusateri AE, Moore EE, Hassoune A, Chapman MP, Daley BJ, Miller RS, Harbrecht BG, Claridge JA, Phelan HA, Brown JB, Zuckerbraun BS, Neal MD, Yazer MH, Sperry JL (2020) Prehospital plasma in injured patients is associated with survival principally in blunt injury: results from two randomized prehospital plasma trials. J Trauma Acute Care Surg 88(1):33–41. 10.1097/TA.0000000000002485 31524836 10.1097/TA.0000000000002485 PMC6923541

[76] Pusateri AE, Moore EE, Moore HB, Le TD, Guyette FX, Chapman MP, Sauaia A, Ghasabyan A, Chandler J, McVaney K, Brown JB, Daley BJ, Miller RS, Harbrecht BG, Claridge JA, Phelan HA, Witham WR, Putnam AT, Sperry JL (2020) Association of prehospital plasma transfusion with survival in trauma patients with hemorrhagic shock when transport times are longer than 20 minutes: a post hoc analysis of the PAMPer and COMBAT clinical trials. JAMA Surg 155(2):e195085. 10.1001/jamasurg.2019.5085 31851290 10.1001/jamasurg.2019.5085 PMC6990948

[77] Gruen DS, Guyette FX, Brown JB, Okonkwo DO, Puccio AM, Campwala IK, Tessmer MT, Daley BJ, Miller RS, Harbrecht BG, Claridge JA, Phelan HA, Neal MD, Zuckerbraun BS, Yazer MH, Billiar TR, Sperry JL (2020) Association of Prehospital Plasma With Survival in Patients With Traumatic Brain Injury: A Secondary Analysis of the PAMPer Cluster Randomized Clinical Trial. JAMA Netw Open 3(10):e2016869. 10.1001/jamanetworkopen.2020.16869 33057642 10.1001/jamanetworkopen.2020.16869 PMC7563075

[78] Guyette FX, Sperry JL, Peitzman AB, Billiar TR, Daley BJ, Miller RS, Harbrecht BG, Claridge JA, Putnam T, Duane TM, Phelan HA, Brown JB (2021) Prehospital blood product and crystalloid resuscitation in the severely injured patient: a secondary analysis of the prehospital air medical plasma trial. Ann Surg 273(2):358–364. 10.1097/SLA.0000000000003324 30998533 10.1097/SLA.0000000000003324

[79] Thies KC, Ruetzler K (2022) Prehospital blood transfusion: who benefits? Lancet Haematol 9(4):e238–e239. 10.1016/S2352-3026(22)00074-6 35271809 10.1016/S2352-3026(22)00074-6

[80] Ziegler B, Bachler M, Haberfellner H, Niederwanger C, Innerhofer P, Hell T, Kaufmann M, Maegele M, Martinowitz U, Nebl C, Oswald E, Schöchl H, Schenk B, Thaler M, Treichl B, Voelckel W, Zykova I, Wimmer C, Fries D (2021) Efficacy of prehospital administration of fibrinogen concentrate in trauma patients bleeding or presumed to bleed (FIinTIC): a multicentre, double-blind, placebo-controlled, randomised pilot study. Eur J Anaesthesiol 38(4):348–357. 10.1097/EJA.0000000000001366 33109923 10.1097/EJA.0000000000001366 PMC7969176

[81] Tucker H, Brohi K, Tan J, Aylwin C, Bloomer R, Cardigan R, Davenport R, Davies ED, Godfrey P, Hawes R, Lyon R, McCullagh J, Stanworth S, Thompson J, Uprichard J, Walsh S, Weaver A, Green L (2023) Association of red blood cells and plasma transfusion versus red blood cell transfusion only with survival for treatment of major traumatic hemorrhage in prehospital setting in England: a multicenter study. Crit Care 27(1):25. 10.1186/s13054-022-04279-4 36650557 10.1186/s13054-022-04279-4 PMC9847037

[82] Levy MJ et al (2024) Implementation of a prehospitalwhole blood program: lessons learned. J Am Coll Emerg Physicians Open 5(2):e13142. 10.1002/emp2.13142 38524357 10.1002/emp2.13142 PMC10958095

[83] Standl T, Annecke T, Cascorbi I, Heller AR, Sabashnikov A, Teske W (2018) The nomenclature, definition and distinction of types of shock. Dtsch Ärztebl Int 115:757–768. 10.3238/arztebl.2018.0757 30573009 10.3238/arztebl.2018.0757 PMC6323133

[84] Jänig C, Willms C, Schwietring J, Güsgen C, Willms A, Didion N, Gruebl T, Bieler D, Schmidbauer W (2023) Patients at risk for transfusion—a six-year multicentre analysis of more than 320,000 helicopter emergency medical service missions. J Clin Med 12(23):7310. 10.3390/jcm12237310 38068362 10.3390/jcm12237310 PMC10706994

[85] Maegele M, Lier H, Hossfeld B (2023) Pre-hospital blood products for the care of bleeding trauma patients. Dtsch Ärztebl Int 120(40):670–676. 10.3238/arztebl.m2023.0176 37551452 10.3238/arztebl.m2023.0176 PMC10644958

[86] Jenkins D et al (2014) Implementation and execution of civilian remote damage control resuscitation programs. Shock 41(1):84–89. 10.1097/SHK.0000000000000133 24662783 10.1097/SHK.0000000000000133

[87] Reifferscheid F (2022) Wissenschaftliche Evaluation zum Einsatz von Blutprodukten im Rettungsdienst. Notarzt 38:292

